# A new species of the genus *Nihonotrypaea* Manning & Tamaki, 1998 (Crustacea, Decapoda, Axiidea, Callianassidae) from the South China Sea

**DOI:** 10.3897/zookeys.457.8198

**Published:** 2014-11-25

**Authors:** Wenliang Liu, Ruiyu Liu

**Affiliations:** 1 Shanghai Key Lab for Urban Ecological Processes and Eco-Restoration, School of Ecological and Environmental Science, East China Normal University, Shanghai 200062, China; 2 Institute of Oceanology, Chinese Academy of Sciences, Qingdao 266071, China (Deceased 16 July 2012)

**Keywords:** Callianassidae, *Nihonotrypaea*, new species, South China Sea

## Abstract

A new species of the genus *Nihonotrypaea* Manning & Tamaki, 1998, *Nihonotrypaea hainanensis* **sp. n.**, collected from the South China Sea, is described and illustrated. It is distinguishable from *Nihonotrypaea harmandi* (Bouvier, 1901), *Nihonotrypaea japonica* (Ortmann, 1891), *Nihonotrypaea thermophila* Lin, Komai & Chan, 2007 and *Nihonotrypaea makarovi* Martin, 2013 by having the elongated carpus of the male and female major cheliped. The new species is distinguishable from *Nihonotrypaea petalura* (Stimpson, 1860) by the proximolower margin of the carpus of the male major cheliped bearing several small denticles.

## Introduction

While working on the taxonomic study of the axiidean fauna (Crustacea, Decapoda) of the China Sea, an undescribed species assignable to the genus *Nihonotrypaea* Manning & Tamaki, 1998 was found from the intertidal sand flat of Hainan Province, South China Sea. The genus *Nihonotrypaea* is characterized by the following characters: carapace lacking rostral spine, minute median terminal spinule present or absent; antennular and antennal peduncles subequal in length; third maxilliped lacking exopod, ischium-merus operculiform, distal margin of merus slightly projecting beyond articulation with carpus; chelipeds unequal, both with lobe-like protrusions on lower margin of merus; first pleopod slender, uniramous in both sexes; second pleopod absent in male, slender, biramous in female; third to fifth pleopods with stubby, slightly projecting appendix internae in both sexes (Manning and Tamaki 1998, Lin et al. 2007).

Five species are known, all from the northwestern Pacific: *Nihonotrypaea harmandi* (Bouvier, 1901), *Nihonotrypaea japonica* (Ortmann, 1891), *Nihonotrypaea petalura* (Stimpson, 1860), *Nihonotrypaea thermophila* Lin, Komai & Chan, 2007 and *Nihonotrypaea makarovi* Martin, 2013. The status of the genus has been subject to disagreement. Sakai (1999, 2005) treated *Nihonotrypaea* as a synonym of *Callianassa* Leach, 1814. Subsequently, Sakai (2011) redefined several genera belonging to the subfamily Callianassinae, and placed *Nihonotrypaea* under the synonymy of *Trypaea* Dana, 1852. On the other hand, *Nihonotrypaea* was recognized as a valid genus by Tudge et al. (2000), Lin et al. (2007) and Martin (2013). Preliminary molecular phylogenetic analyses of the family Callianassidae (Felder and Robles 2009) showed that the representatives of the genus *Nihonotrypaea* grouped together with those of the genus *Neotrypaea* in a strongly supported monophyletic clade, encompassing somewhat less supported subclades that do not clearly resolve the status of *Nihonotrypaea*. Meanwhile, the specific status of *Nihonotrypaea harmandi* has been subject of debate: for example, Tamaki et al. (1997, 1999), Tamaki and Miyabe (2000), Wardiatno and Tamaki (2001), Tamaki and Harada (2006) and Lin et al. (2007) considered it a valid species. On the other hand, Sakai (1969, 1987, 1999, 2004, 2011) considered it as a junior synonym of *Nihonotrypaea japonica* or *Trypaea japonica*.

In this study, we provisionally recognize the genus *Nihonotrypaea* according to the latest literature (Martin 2013), and the higher classification of Callianassidae is not discussed in depth, as it is beyond the scope of this paper. Here we describe and illustrate a new species, referred to *Nihonotrypaea* from the Chinese seas.

## Methods

All specimens examined have been deposited in the Institute of Oceanology, Chinese Academy of Sciences, Qingdao, China (IOCAS). The drawings were made with the aid of drawing tube mounted on a Zeiss Stemi Sv11 compound microscope. The following abbreviation is used throughout the text: CL: carapace length.

## Taxonomy

### Family Callianassidae Dana, 1852 Genus *Nihonotrypaea* Manning & Tamaki, 1998

#### Nihonotrypaea hainanensis sp. n.

Taxon classificationAnimaliaDecapodaCallianassidae

http://zoobank.org/4940C2F3-FB80-4981-B45D-6FD7726E8DEA

Figs 1 , 2 , 3

##### Material examined.

Holotype, adult male (CL 2.9 mm), MBM136863, Sanya City, Hainan Province, South China Sea, 18°17.2'N, 109°26.4'E, intertidal zone, sand, coll. Fengxuan Zhang, 5 April 1958. Paratypes, 2 ovig. females (CL 3.0, 3.2 mm), MBM136863, same data as holotype.

##### Description.

**Figure 1.**
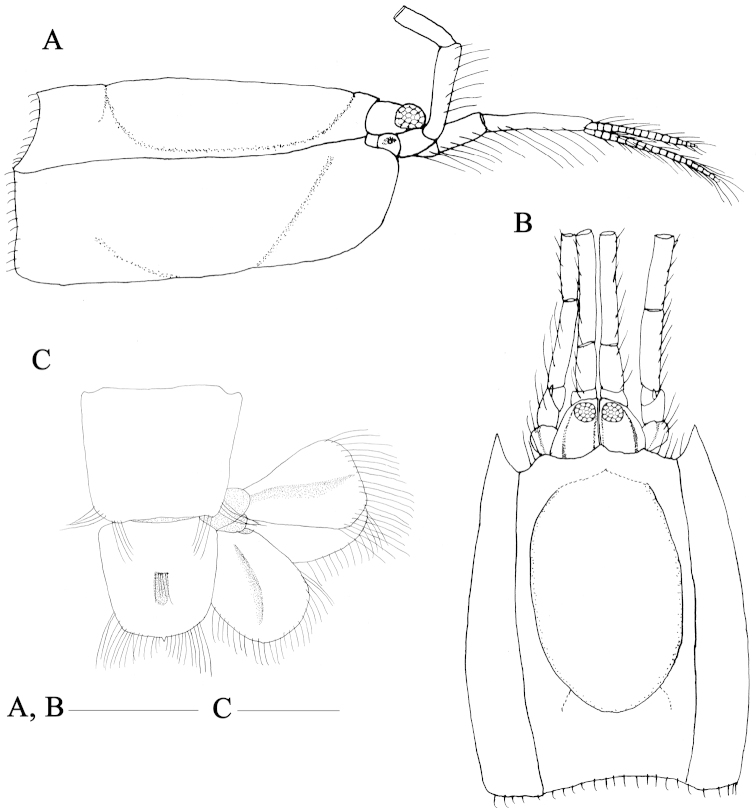
*Nihonotrypaea hainanensis* sp. n. Holotype male, MBM136863. **A–B** carapace, dorsal and lateral view **C** pleomere 6, telson and uropods, dorsal view. Scale = 1 mm.

Rostrum (Fig. 1A, B) broadly triangular, obtuse, directed slightly downwards, reaching proximal 0.2 length of eyestalk in dorsal view. Carapace smooth (Fig. 1A, B), approximately 0.3 of total body length; dorsal oval well defined, 0.74 as long as carapace. Cervical groove located at posterior quarter; linea thalassinica complete.

Eyestalks (Fig. 1A, B) moderately long, sub-triangular in dorsal view, lateral margin distinctly sinuous, anterolateral margin forming thin ridge extending to rounded terminal margin, reaching 4/5 length of first article of antennular peduncle; cornea subterminal, disk-shaped, swollen in lateral view, with scattered brown-pigmented spots, corneal width less than half of basal width of eyestalk.

Antennular peduncle (Fig. 1A, B) subequal in length to antennal peduncle; third article 2.4 times as long as second article; outer flagellum and inner flagellum shorter than peduncle. Antennal peduncle with rudimentary scale on article 3; fifth article distinctly shorter than fourth article.

**Figure 2.**
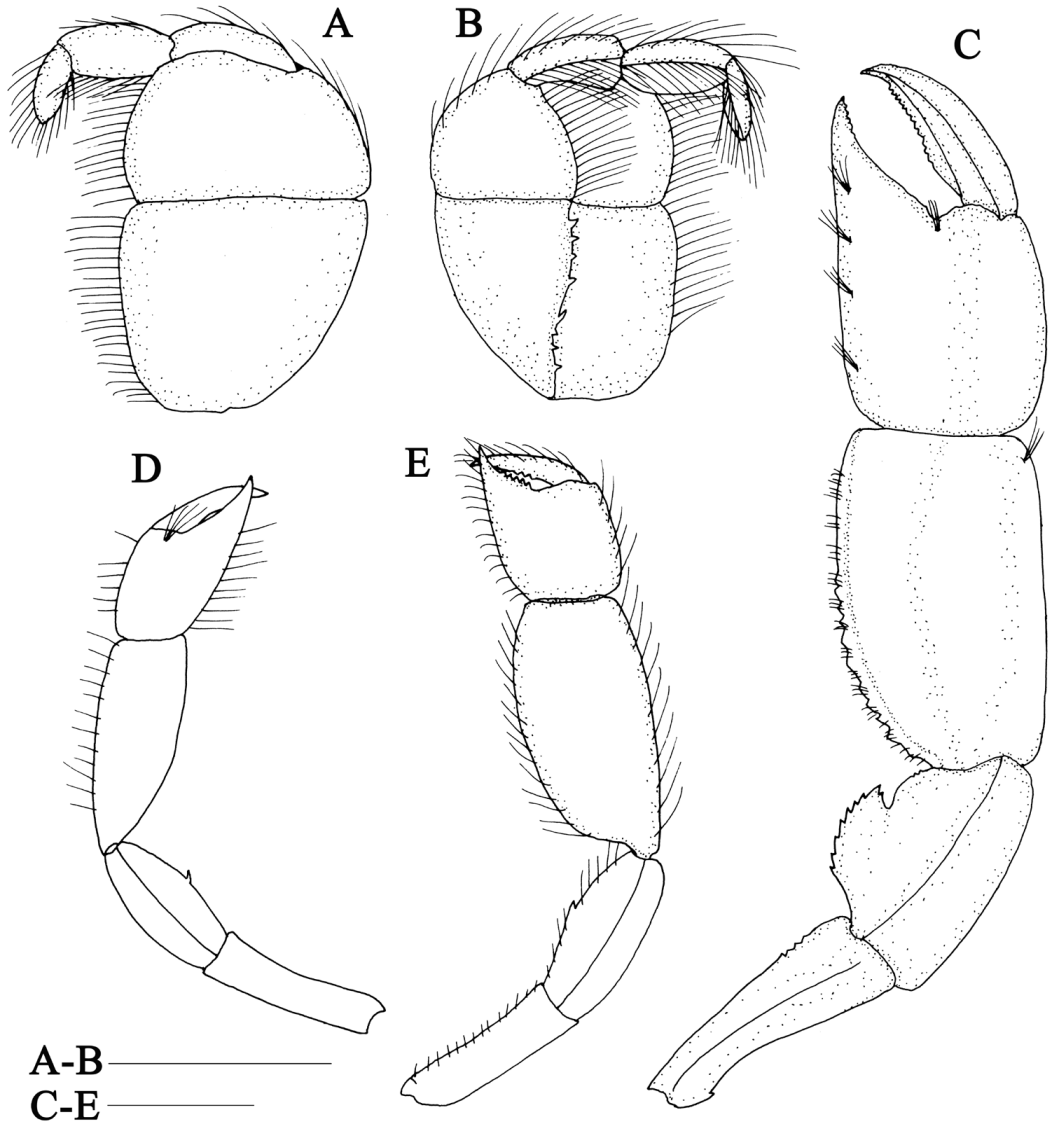
*Nihonotrypaea hainanensis* sp. n. **A–D** Holotype male, MBM136863 **E** Paratype female, MBM136863. **A–B** maxilliped 3, outer and inner views **C** male left major cheliped, outer view **D** male right minor cheliped, outer view **E** female left major cheliped, outer view. Scale = 1 mm.

Third maxilliped (Fig. 2A, B) without exopod; ischium-merus operculiform, with dense setae on ventral margin; ischium broader than long, inner surface with crista dentata consisting of slightly sinuous row of small and sharp denticles; merus broader than long and approximately 0.7 times as long as ischium; carpus cup-shaped, shorter than merus; propodus slender, almost as long as carpus; dactylus moderately slender, digitiform, shorter than propodus. Inner surface of merus to dactylus with blunt longitudinal ridge bearing rows of long setae.

Pereopods 1 unequal and strongly dissimilar. Male major cheliped massive (Fig. 2C). Ischium 3.0 times as long as high; upper margin sinuous, with 1 small denticle proximally; lower margin slightly convex, armed with 4 inconspicuous denticles in distal 0.3. Merus as long as ischium, 1.9 times as long as high; upper margin slightly convex, unarmed; lower margin sinuous, with prominent lobe-like process proximally and 2 minute, inconspicuous denticles subdistally; lobe-like process terminating in acutely, with 7 sharp denticles except for the tip on ventral margin and 1 subterminal denticle distally. Carpus elongated, 1.7 times as long as high, 1.3 times as long as merus; upper margin almost straight; proximolower margin gently convex, with row of several small, blunt denticles. Chela heavy, approximately 1.6 times as long as high; palm subquadrate, approximately 1.1 times as long as high, 0.6 times as long as carpus; fixed finger 0.5 times as long as palm, cutting edge with some inconspicuous denticles in distal 0.3; dactylus slightly curved distally, slightly longer than fixed finger, cutting edge sinuous, entire in the distal 0.2 and proximal 0.3, and armed with row of denticles on middle.

Male minor cheliped (Fig. 2D) with ischium 3.5 times as long as high, unarmed. Merus 0.9 times as long as ischium, about 2.3 times as long as high, upper and lower margin slightly convex, unarmed; outer surface medially swollen. Carpus 2.5 times as long as high, 1.6 times as long as merus, abruptly narrowed at base; upper margin almost straight; proximolower margin convex. Chela gapped between slender dactylus and fixed finger; palm nearly as long as high, 0.5 times as long as carpus; fixed finger tapering distally to acute tip, cutting edge unarmed; dactylus slightly longer as palm, slender, unarmed on concave cutting edge.

Female major cheliped (Fig. 2E) with ischium 3.5 times as long as high, upper margin almost straight; lower margin slightly concave, unarmed. Merus almost as long as ischium, upper margin slightly convex, unarmed; lower margin slightly convex, with small tooth at midlength. Carpus 2.0 times as long as high, 1.4 times as long as merus; upper margin almost straight. Chela similar to that of male but relatively smaller; palm subquadrate, almost as long as high; fixed finger 0.5 times as long as palm, cutting edge with some small denticles on proximal half; dactylus slightly longer than fixed finger, cutting edge sinuate and with some small denticles on middle. Minor cheliped in female similar to that of male, and about 0.8 times as long as major cheliped.

**Figure 3.**
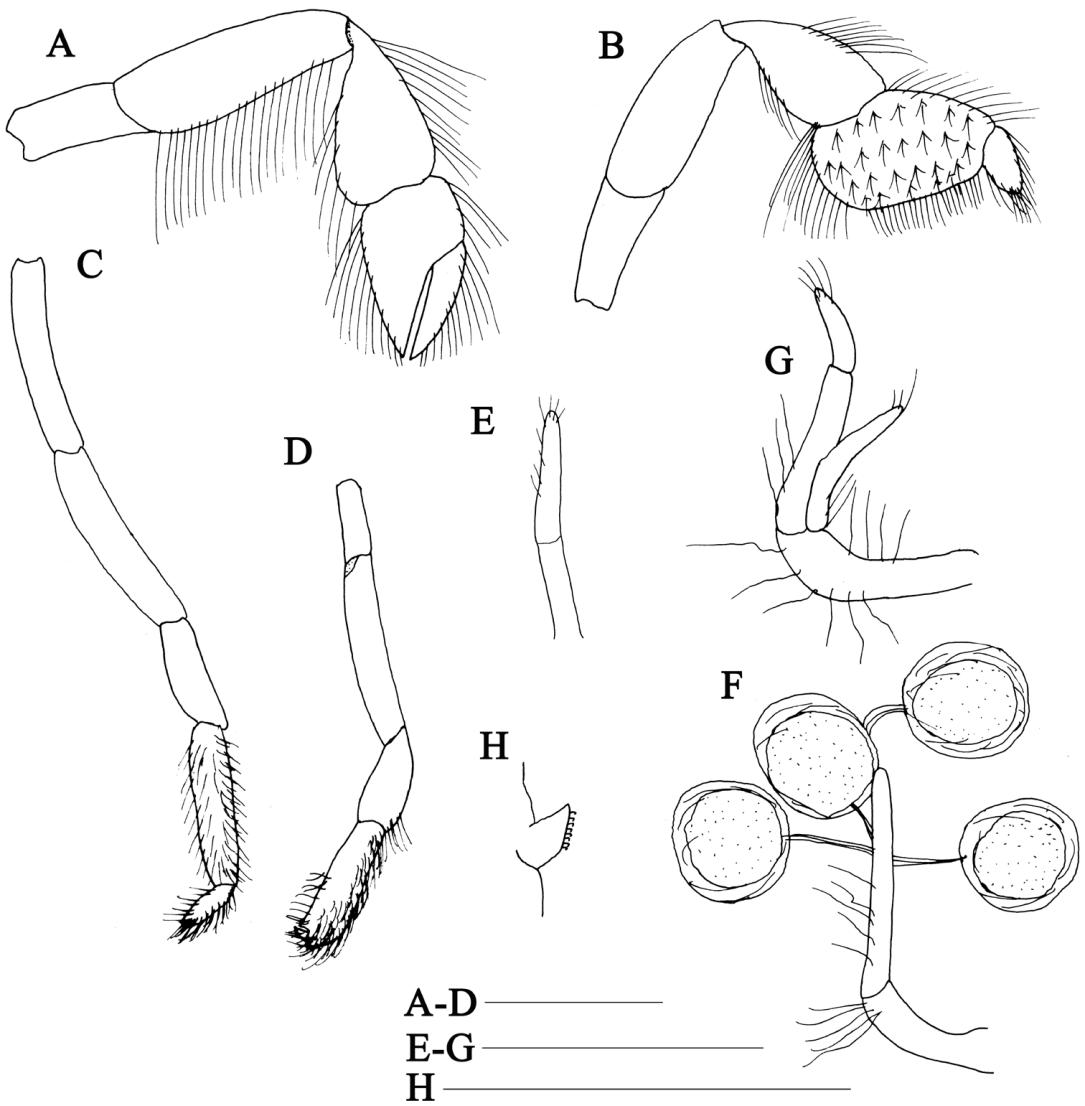
*Nihonotrypaea hainanensis* sp. n. **A–E** Holotype male, MBM136863 **F–H** Paratype female, MBM136863 **A–D** pereopods 2–5, outer views **E** male pleopod 1, posterior view **F** female pleopod 1, posterior view **G** female pleopod 2, posterior view **H** appendix internae of pleopod 3, posterior view. Scale = 1 mm.

Pereopod 2 (Fig. 3A) chelate. Ischium 2.0 times as long as high; merus about 3.0 times as long as high, upper margin smooth, lower margin protruding and with row of dense long setae; carpus subtriangular, shorter than merus; chela slightly shorter than carpus, with dense setae on lower and upper margins; palm with upper margin slightly convex; dactylus 1.9 times as long as upper margin of palm; carpus and chela fringed with short to long setae along margins.

Pereopod 3 (Fig. 3B) simple, moderately slender. Ischium slender, approximately 2.0 times as long as high; merus approximately 2.7 times as long as high; carpus subtriangular, shorter and broader than merus, broadest subdistally, approximately 1.9 times as long as high; propodus subrectangular, lower margin roundly swollen, upper margin slightly convex and 0.6 length of carpus, with numerous tufts of setae on lateral surface and row of thick setae along upper and lower margin, no distinct heel delimited; dactylus subtriangular, upper and lower margin convex, outer surface densely setose, terminating in corneous tip.

Pereopod 4 (Fig. 3C) slender, all articles unarmed. Ischium rectangular; merus slightly longer than ischium; carpus 0.6 length of merus; propodus 2.8 times as long as carpus, lower margin densely setose; dactylus tapering distally, setose on lateral margin.

Pereopod 5 (Fig. 3D) slender, semichelate, all articles unarmed. Ischium rectangular; merus about 2.5 as long as ischium; carpus about 0.6 length of merus, upper margin swollen; propodus about 1.3 times as long as carpus, lower distal corner projecting to form a chela with dactylus, lateral surface beset distally with dense setae; dactylus hooked toward external side of fixed finger, tips of dactylus and fixed finger obtuse.

Pleomeres smooth dorsally. Pleomere 1 narrowing anteriorly in dorsal view; dorsal tergite fused with the lateral pleurites; pleuron weakly developed but with clearly defined ventral margin. Pleomere 2 distinctly longer than other pleomeres, with posterolateral margin of pleuron slightly expanded, bearing lateral row of plumose setae. Pleomere 3–5 with pleura each having tuft of moderately long plumose setae. Pleomere 6 (Fig. 1C) subquadrate in dorsal view, very slightly narrowed posteriorly; lateral margin smooth, without conspicuous notch.

Telson (Fig. 1C) trapezoidal, almost as long as wide and 0.8 times as long as pleomere 6; dorsal surface convex with a short transverse row of setae medially; lateral margin unarmed; posterior margin gently convex, with small median spine.

Male pleopod 1 (Fig. 3E) uniramous, 2-articulated; distal article with some distal setae. Male pleopod 2 absent. Female pleopod 1 (Fig. 3F) uniramous, 2-articulated; protopod article sinuous, shorter than ramus; ramus spatulate distally and weakly thickened basally, bearing short setae on both margins. Female pleopod 2 (Fig. 3G) biramous; exopod 2-articulated, approximately 1.5 times as long as endopod, sinuous, bearing some short setae distally; endopod with long setae on proximal part and short setae terminally. Pleopods 3–5 biramous, foliaceous, rami broad; appendix internae (Fig. 3H) stubby, slightly projecting beyond margin of endopod, bearing numerous small adhesive hooks along mesial margin.

Uropodal endopod (Fig. 1C) subovate, slightly longer than telson, 1.5 times as long as wide; margins unarmed; with distinct submedian carina on dorsal surface. Uropodal exopod (Fig. 1C) broadened and fan-shaped, almost as long as wide; margins unarmed, with a distinct submedian carina on dorsal surface.

##### Remarks.

*Nihonotrypaea hainanensis* sp. n. is the sixth species assigned to the genus. The new species is closely related to *Nihonotrypaea harmandi* (Bouvier, 1901), *Nihonotrypaea japonica* (Ortmann, 1891) and *Nihonotrypaea petalura* (Stimpson, 1860) in the antennular peduncle being subequal in the length to the antennal peduncle, whereas *Nihonotrypaea thermophila* Lin, Komai & Chan, 2007 and *Nihonotrypaea makarovi* Martin, 2013 have the antennular peduncle being slightly to distinctly shorter than the antennal peduncle. *Nihonotrypaea hainanensis* sp. n. is distinguishable from *Nihonotrypaea harmandi* (Bouvier, 1901), *Nihonotrypaea japonica* (Ortmann, 1891), *Nihonotrypaea thermophila* Lin, Komai & Chan, 2007 and *Nihonotrypaea makarovi* Martin, 2013 by having a relatively long carpus (approximately 1.7 times as long as high in male and 2.0 times as long as high in female) of the major cheliped (versus carpus subquadrate, in male, approximately 1.1 times as long as high in the later four species; in female approximately 1.5 time as long as high in *Nihonotrypaea makarovi*, 1.1 times as long as high in *Nihonotrypaea harmandi*, *Nihonotrypaea japonica* and *Nihonotrypaea thermophila*). Sakai (1969) discussed the variation of the major cheliped in *Nihonotrypaea petalura*: some specimen have the oblong carpus (approximately 1.5–1.8 times as long as high), which is similar to *Nihonotrypaea hainanensis* sp. n., but the new species can be readily distinguished from *Nihonotrypaea petalura* by the proximolower margin of the carpus of the major cheliped bearing several small denticles (versus smooth in *Nihonotrypaea petalura*).

It is worth mentioning that considerable similarities were also found between species assigned to *Nihonotrypaea* and those of *Biffarius* Manning & Felder, 1991, especially regarding operculiform ischium-mems on the third maxilliped. The new species is also closely related to *Biffarius ceramicus* (Fulton & Grant, 1906) and *Biffarius melissae* Poore, 2008 in having a broadly triangular rostrum and the antennular peduncle being subequal in length to the antennal peduncle. It is, however, distinguishable from *Biffarius melissae* Poore, 2008 by having the telson almost as long as wide (telson about 0.8 times as long as wide), and can be distinguished from *Biffarius ceramicus* (Fulton & Grant, 1906) by having a relatively long carpus of the male major cheliped (approximately 0.8 times as long as high).

##### Etymology.

The species name is based on the type locality, Hainan Province of China.

##### Distribution and habitat.

Presently only known from the type locality. Found in the intertidal zone in sand.

## Supplementary Material

XML Treatment for Nihonotrypaea hainanensis
